# Monotonic and Cyclic Loading/Unloading Tensile Behavior of 3D Needle-Punched C/SiC Ceramic-Matrix Composites

**DOI:** 10.3390/ma14010057

**Published:** 2020-12-24

**Authors:** Yufeng Liu, Longbiao Li, Zhongwei Zhang, Xiang Xiong

**Affiliations:** 1 Science and Technology of Advanced Functional Composite Materials Laboratory, Aerospace Research Institute of Materials & Processing Technology, Beijing 100076, China; liuyf123@csu.edu.cn; 2 Powder Metallurgy Research Institute, Central South University, Changsha 410083, China; xiongx@csu.edu.cn; 3 College of Civil Aviation, Nanjing University of Aeronautics and Astronautics, No.29 Yudao St., Nanjing 210016, China; 4 Institute of Advanced Structure Technology, Beijing Institute of Technology, Beijing 100081, China; 6120190123@bit.edu.cn

**Keywords:** ceramic-matrix composites (CMCs), needle-punched, C/SiC, tensile, hysteresis, matrix cracking, interface debonding, fibers pullout

## Abstract

In this paper, monotonic and cyclic loading/unloading tensile behavior of four different 3D needle-punched C/SiC composites are investigated. Under tensile loading, multiple micro parameters of tensile tangent modulus, tensile strength, and fracture strain are used to characterize tensile damage and fracture behavior. Under cyclic loading/unloading, multiple damage micro parameters of unloading residual strain, tensile peak strain, hysteresis loops width, hysteresis loops area, unloading and reloading inverse tangent modulus (ITM) are used to describe the tensile damage evolution. After tensile fracture, fracture surfaces were observed under a scanning electron microscope (SEM). Damage of matrix cracking, interface debonding, fibers fracture and pullout in different plies is observed. Relationships between composite tensile mechanical behavior, damage parameters, and micro damage mechanisms are established. When the fiber volume fraction along the loading direction increases, the composite initial tangent modulus, tensile strength and fracture strain increase, and the unloading residual strain, peak strain, hysteresis width and hysteresis area decrease. For Types 1–4 3D needle-punched C/SiC composite, the fiber volume lies in the range of 25.6–32.8%, the composite initial tangent modulus was in the range of 161.4–220.4 GPa, the composite tensile strength was in the range of 64.4–112.3 MPa, and the composite fracture strain was in the range of 0.16–0.25%.

## 1. Introduction

Carbon fiber-reinforced silicon carbide ceramic-matrix composites (C/SiC CMCs) have the characteristics of high temperature resistance, high strength, low density, low thermal expansion coefficient, good thermal conductivity and corrosion resistance, have better oxidation resistance than C/C composites, and have become the first choice of high-temperature structural materials [1]. C/SiC composites have broad application prospects in advanced propulsion systems and thermal protection systems (TPS) of reentry vehicles [2].

As a reinforced skeleton of composite materials, carbon fiber preform has a decisive impact on the properties of the materials. Three-dimensional preform contains load-bearing fibers in different directions, which overcome the shortcomings of low damage tolerance and weak interlaminar performance of two-dimensional preform, and show higher bearing performance [3]. The traditional three-dimensional carbon fiber forming technology, such as braiding, weaving and knitting, is often complex and costly, and is not easy to produce in batches. To improve the interlaminar debonding resistance, a needled preform, Noveltex, was developed by the SEP (Snecma Propulsion Solide) company at the end of the 1970s, which can efficiently produce needle punched carbon fiber preforms with various shapes [4,5,6,7]. The needling technology uses the needle to stab the carbon fiber cloth, short-chopped-fiberweb and other fiber composite materials, and introduces part of the in-plane fiber into the ply thickness direction to generate vertical fiber clusters so that the carbon fiber and short-chopped-fiberweb are closely combined to form a preform with certain strength in the plane and between layers. The needle punched products with arbitrary shape and size, variable cross-section shape, and variable density can be prepared by adjusting the process parameters [8].

Many researchers performed theoretical and experimental investigations on mechanical behavior of needle-punched CMCs. In the experimental research area, Liu et al. [9] investigated the effects of needle punching depth and density on mechanical properties of composite fabric. The density of composite fabric increases with the increase of needle punching depth, and the tensile strength of composite fabric in the X-Y direction decreases with the increase of needle punching density. Yang et al. [10] investigated the chemical vapor infiltration (CVI) fabrication process of a needle-punched C/C composite. In the initial stage of the CVI process, pyrolytic carbon (PyC) began to deposit on the surface of carbon fiber and forms interphase. As the deposition process continued, the PyC began to fill the pores in the needle-punched preform. Compared with the liquid-phase impregnation method, the pores produced by CVI are mainly distributed in the matrix, while many pores are formed between the carbon matrix and the carbon fiber by the liquid-phase impregnation process, which reduce the interfacial bonding strength between the matrix and the fiber and affect the mechanical properties of C/C composites [11]. Guo et al. [12] investigated the mechanical properties of 3D needle-punched T700^TM^ C/SiC composite at room temperature. The needling fibers interlined the layers along the thickness direction and improved the interlaminar properties of the composite. The interlaminar shear strength of 3D needle-punched C/SiC was approximately 95 MPa, which is much higher than that of 2D woven C/SiC composite, i.e., 35 MPa. The tensile and flexural strength were approximately 159 and 350 MPa, respectively. Nie et al. [13] investigated the loading/unloading tensile behavior of 3D needle-punched T300^TM^ C/SiC composite at room temperature. The composite tensile strength and fracture strain were approximately 129.6 MPa and 0.61%. Non-linear tensile behavior of 3D needle-punched C/SiC is mainly attributed to matrix cracking and propagation, debonding and slip at the interface, and fracture and pullout of the fibers. When the unloading stress is lower than 80 MPa, the residual strain and unloading modulus increase linearly with increasing unloading stress. However, when the unloading stress is higher than 80 MPa, the residual strain and unloading modulus increase non-linearly with unloading stress. Chen et al. [14] investigated high temperature tensile mechanical properties of 3D needle-punched C/SiC composites. The composite tensile strength increases gradually from approximately 98.7 MPa at room temperature to approximately 162.6 MPa at 1800 °C and decreases to approximately 154.3 MPa at 2000 °C. At elevated temperature, a large amount of fiber was pulled out, indicating that the interfacial shear stress decreased with increase of temperature. Liu et al. [15] investigated the mechanical properties of 3D needle-punched CVI C/SiC bolts. At room temperature, the tensile strength and shear strength of needle-punched C/SiC bolts were approximately 151.7 MPa and 85.6 MPa. The tensile performance of 3D needle-punched C/SiC bolts is lower than that of 2D woven C/SiC bolts, and the shear properties of 3D needle-punched C/SiC bolts are better than that of 2D woven C/SiC bolts. On the fracture surface of 3D needle-punched C/SiC bolts, broken fibers were pulled out out of the matrix, and exhibited step fracture morphology. In the theoretical research area, Xie et al. [16] investigated the shear damage behavior of 3D needle-punched C/SiC composite. A plasticity-damage nonlinear constitutive model was established to predict the non-linear shear behavior of the composite. The shear non-linearity of the composite was induced by inner damages of matrix cracking and interface debonding. Li [17] developed micromechanical methods to predict the tensile damage and fracture of 2D and 2.5D C/SiC composites at room temperature, and established the relationships between internal damage inside composites and the nonlinear stress–strain relationship. Li [18] considered the effects of the interface oxidation and fibers fracture on tensile stress–strain curves of unidirectional mini-CMCs at elevated temperature, and analyzed stochastic loading on tensile stress–strain curves of different CMCs at room temperature [19]. Callaway and Zok [20], Li et al. [21], and Guo et al. [22] performed theoretical investigations on the tensile damage fracture process of SiC/SiC minicomposite and 2D SiC/SiC composites at room temperature. The hysteresis loops were analyzed and adopted to characterize the tensile damage of SiC/SiC composites. However, in the research mentioned above, the relationships between tensile nonlinear behavior and internal micro damage evolution of 3D needle-punched C/SiC composites have not been established.

The objective of this paper is to investigate monotonic and cyclic loading/unloading tensile behavior of four different 3D needle-punched C/SiC composites. Under tensile loading, multiple micro damage parameters of tensile tangent modulus, tensile strength and fracture strain are used to characterize tensile damage and fracture. Under cyclic loading/unloading, multiple damage parameters of unloading residual strain, tensile peak strain, hysteresis loops width, hysteresis loops area, unloading and reloading inverse tangent modulus (ITM) are adopted to characterize tensile damage evolution. After tensile fracture, the fracture surfaces are observed under a scanning electron microscope (SEM). Damage of matrix cracking, interface debonding and fiber pullout in different plies is observed and analyzed.

## 2. Materials and Experimental Procedures

The reinforcement of needle-punched composite material is carbon cloth/short-chopped-fiber web needle-punched preform. The preform is composed of carbon fiber non-woven cloth and short-chopped-fiber web layer. The non-woven cloth is composed of unidirectional continuous long fiber bundles, and the fiber web is composed of short carbon fibers randomly distributed in different directions. Automatic production has already been realized in the forming process of needle punched preform, including:

(1) The 0° non-woven cloth, fiber web, and 90° non-woven cloth are alternately stacked.
(2) Needling at the surface of non-woven cloth/fiber web, and during the process of needling, the composite moves horizontally with the conveyor belt, and the needle plate moves up and down at a certain frequency.
(3) Rotating the composite horizontally for 90° and repeat the needling process to ensure the uniformity of the needle holes in the X and Y direction.

Repeat the above three steps until the preform reaches a certain thickness and needling density. In the process of needling, part of the in-plane fiber was introduced into the ply thickness direction to generate vertical fiber clusters, so that the carbon fiber and short-chopped-fiber web are closely combined to form a preform with certain strength in the plane and between layers.

Table 1 shows composite fabric raw material and structure parameters. HTS^TM^ (Toho, Tokyo, Japan) carbon fiber was used in twill woven cloth and T700^TM^ (Toray, Tokyo, Japan) carbon fiber was used in plain woven cloth and short-chopped-fiber web. For HTS^TM^ carbon fiber, the fiber strength is *σ*_fc_ = 4.2 GPa, fiber modulus is *E*_f_ = 240 GPa, fracture strain is *ε*_f_ = 1.8%, and the density is d_f_ = 1790 kg/m^3^. For T700^TM^ carbon fiber, the fiber strength is *σ*_fc_ = 4.9 GPa, fiber modulus is *E*_f_ = 230 GPa, fracture strain is *ε*_f_ = 2.1%, and the density is *d*_f_ = 1790 kg/m^3^. Four different types of fabric preform were introduced for fabricating 3D needled-punched C/SiC composites, as follows:

(1) Type 1, the fabric preform is formed using needle method, and is composed of three layers, including: (a) HTS^TM^-3K twill woven cloth; (b) T700^TM^-12K [±45°] plain woven cloth; (c) T700^TM^-12K short-chopped-fiber web.
(2) Type 2, the fabric preform is formed using needle method and composed of four layers, including: (a) HTS^TM^-3K twill woven ply; (b) T700^TM^-12K [0°] non-woven cloth; (c) T700^TM^-12K [±45°] plain woven cloth; (d) T700^TM^-12K short-chopped-fiber web.
(3) Type 3, the fabric preform is formed using needle method and composed of four layers, including: (a) two layers of HTS^TM^-3K twill woven cloth; (b) T700^TM^ [±45°] plain woven cloth; (c) T700^TM^-12K short-chopped-fiber web.
(4) Type 4, the fabric preform is formed using needle and stitch method and composed of four layers, including: (a) two layers of HTS^TM^-3K twill woven cloth; (b) T700^TM^-12K [±45°] plain woven cloth; (c) T700^TM^-12K short-chopped-fiber web.

In order to improve the surface performance of carbon fibers and release the residual stress of the preform, the needled-punch preform was heat-treated at an elevated temperature of 1800–2000 °C in Ar atmosphere. The density of four original fabric preform were in the range of 540–680 kg/m^3^ and, after being heat-treated, the density of four fabric preform decreased, and was in the range of 460–580 kg/m^3^. The fiber volumes of four original fabric preform were in the range of 30–37.2%, and after being heat-treated, the fiber volumes of four fabric preform decreased and was in the range of 25.6–32.8%. The fiber volume was calculated by:

(1) $$V_{f}=\frac{m_{\mathrm{preform}}}{V_{\mathrm{preform}}\times d_{f}}$$

where *m*_preform_ is the preform mass, *V*_preform_ is the volume of preform, and *d*_f_ is the fiber density.

**Figure 1:**
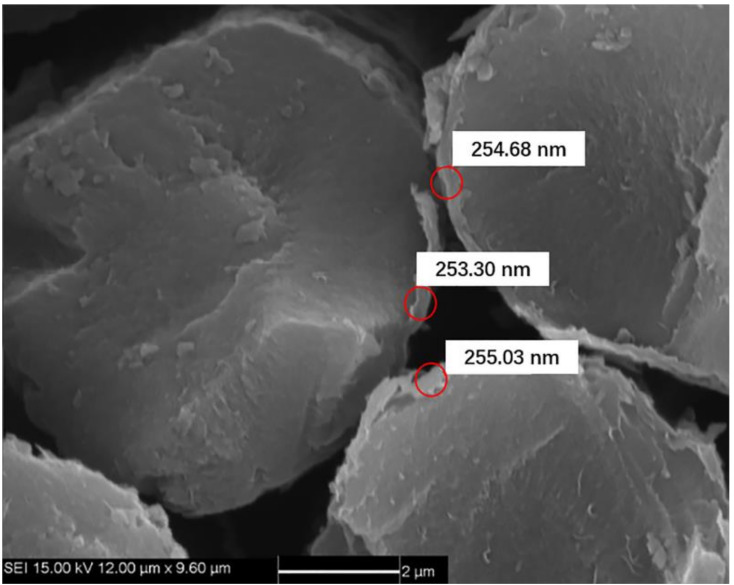
Scanning electron microscope (SEM) micrograph of pyrolytic carbon interface layer on carbon fiber.

**Figure 2:**
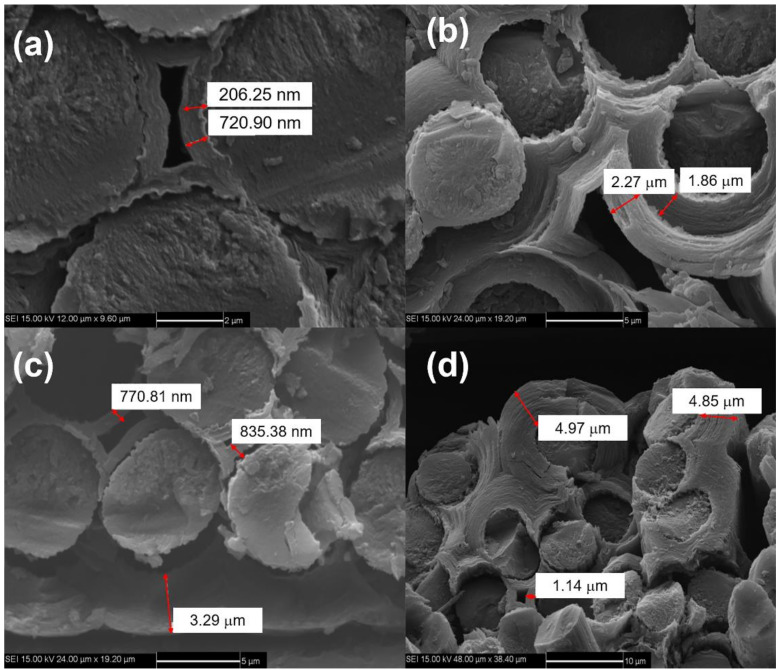
(**a**) First chemical vapor deposition (CVD) carbon matrix; (**b**) second CVD carbon matrix; (**c**) multiple CVD carbon matrix; and (**d**) multiple CVD carbon matrix.

**Figure 3:**
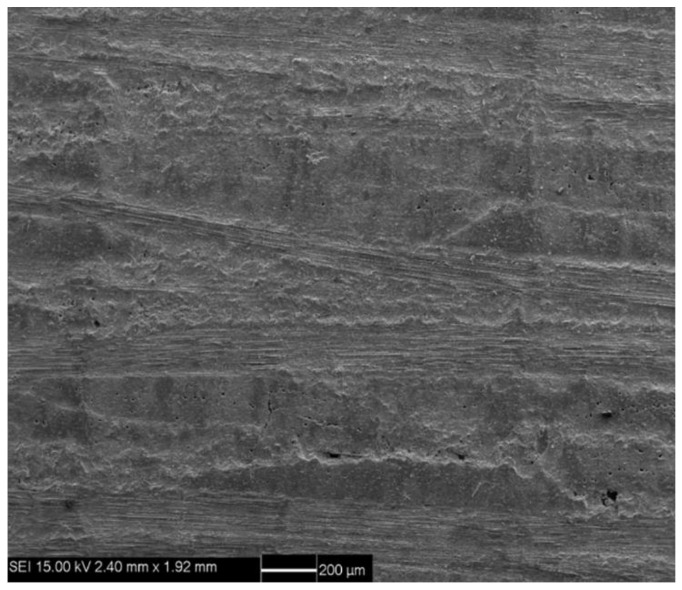
Macroscopic morphology of C/SiC composite after reactive infiltration.

Pyrolytic carbon (PyC) was deposited on the carbon fiber surface as the interphase by the chemical vapor deposition (CVD) process at 850 °C for 20–50 h. The PyC interphase thickness was approximately 200–300 nm, as shown in Figure 1. Using propylene and natural gas as precursor and nitrogen as a carrier diluting gas, the CVD process was carried out for approximately 200–300 h to form the porous C/C composites. The density of porous C/C composites was approximately 1400–1500 kg/m^3^. As the carbon matrix is introduced many times, the carbon matrix was still coated on the surface of carbon fiber monofilament under the low-density state of C/C porous composite, forming a state similar to multi-layer carbon interphase, as shown in Figure 2a,b. With the increase of carbon matrix, the subsequent PyC was coated outside the fiber bundle, as shown in Figure 2c,d. C/SiC composites were prepared by reactive infiltration of Si powder into C/C porous composite. The molten silicon reacted with carbon matrix to form SiC matrix. C/SiC composite was prepared by the co-existence of the C matrix and SiC matrix. Figure 3 shows the macroscopic morphology of C/SiC composite after reactive infiltration.

**Figure 4:**
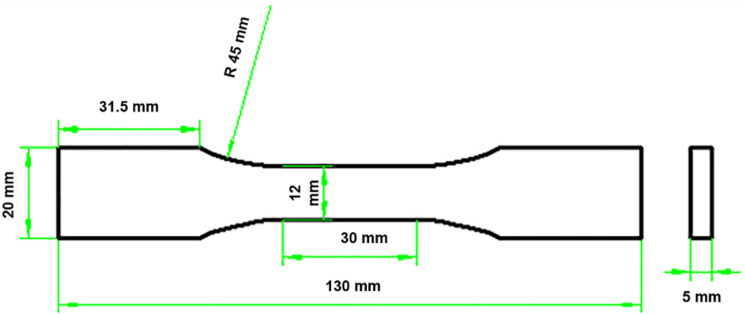
Specimen configuration for monotonic and loading/unloading cyclic tension.

**Figure 5:**
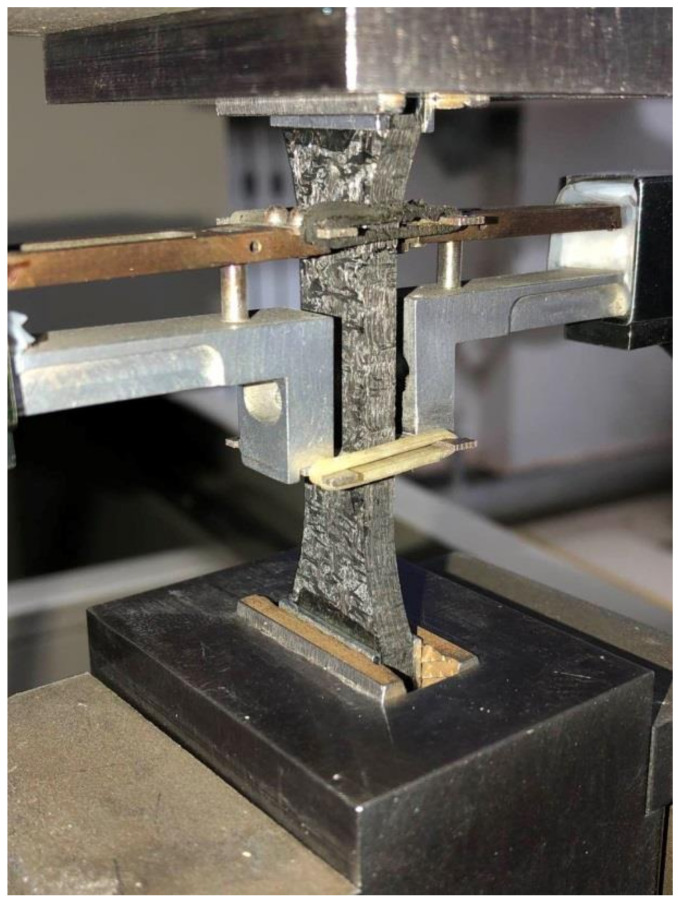
Photograph of tensile specimen and clip-on extensometer.

The dog-bone shaped specimens, with dimensions of 130 mm length, 5 mm thick, and 12 mm width in the gauge section, were cut from 300 mm × 300 mm panels using wire-electrode cutting. Figure 4 shows the specimen size and configurations based on DqES415-2005 standard [23] with 30 mm in the testing gauge length. Li [24,25,26] conducted monotonic and cyclic loading/unloading tensile of unidirectional and cross-ply C/SiC composites at room temperature and 800 °C in air atmosphere. Experimental results were analyzed to characterize the tensile damage and fracture. Monotonic and cyclic loading/unloading tensile tests of 3D needle-punched C/SiC composites were conducted on a SANS CMT5105 testing machine (MTS Systems Corp., Minneapolis, MN, USA) at room temperature. Monotonic and cyclic loading/unloading tensile tests were conducted under displacement control. A clip-on extensimeter was used to obtain the composite strain under monotonic and cyclic loading/unloading strain, as shown in Figure 5. The crosshead speed was 2.0 mm/min for monotonic tensile tests, and 0.5 mm/min for cyclic loading/unloading tensile tests. To analyze failure mechanisms of the composites, the microstructures of the fracture surfaces of the specimens were observed by FEI Quanta 200 field-emission environmental scanning electron microscopy (FEI Ltd., Hillsboro, Oregon, USA). The accelerating voltage was 15 and 20 kV. The vacuum value was set as 3 × 10^−3^ Pa. However, due to the effect of environmental temperature, the vacuum may have changed a little during the process of SEM measurements.

## 3. Experimental Results

**Figure 6:**
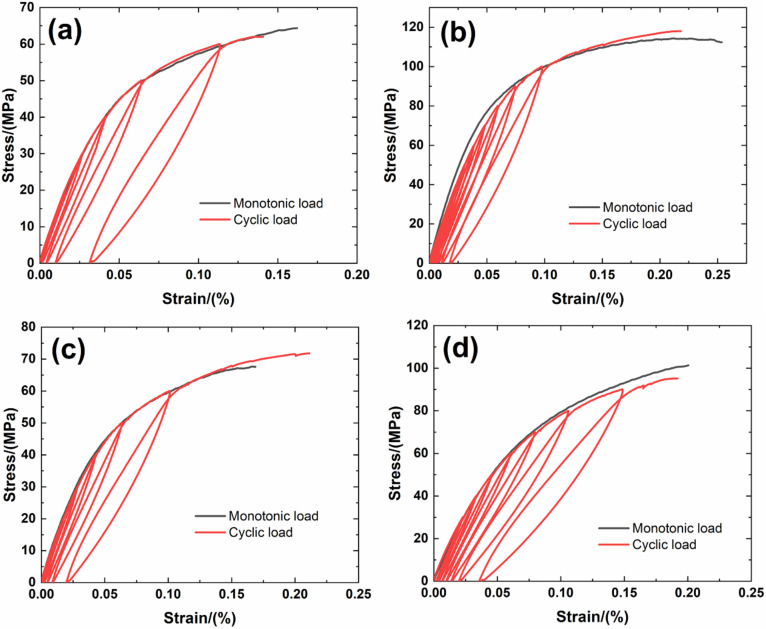
Monotonic and cyclic loading/unloading tensile stress-strain curves corresponding to different 3D needle-punched C/SiC composites with different fabric preforms (**a**) Type 1; (**b**) Type 2; (**c**) Type 3; and (**d**) Type 4.

**Figure 7:**
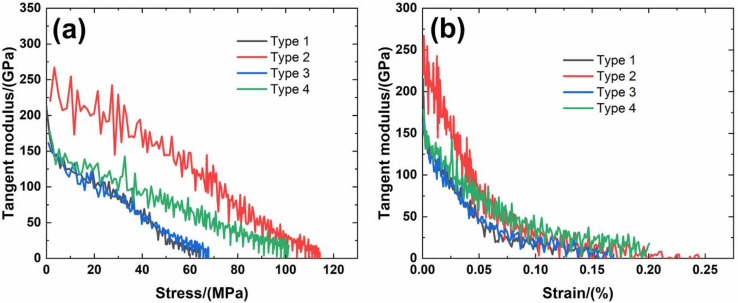
(**a**) Composite tangent modulus versus applied stress curves; and (**b**) composite tangent modulus versus applied strain curves of four different types of 3D needle-punched C/SiC composites.

**Figure 8:**
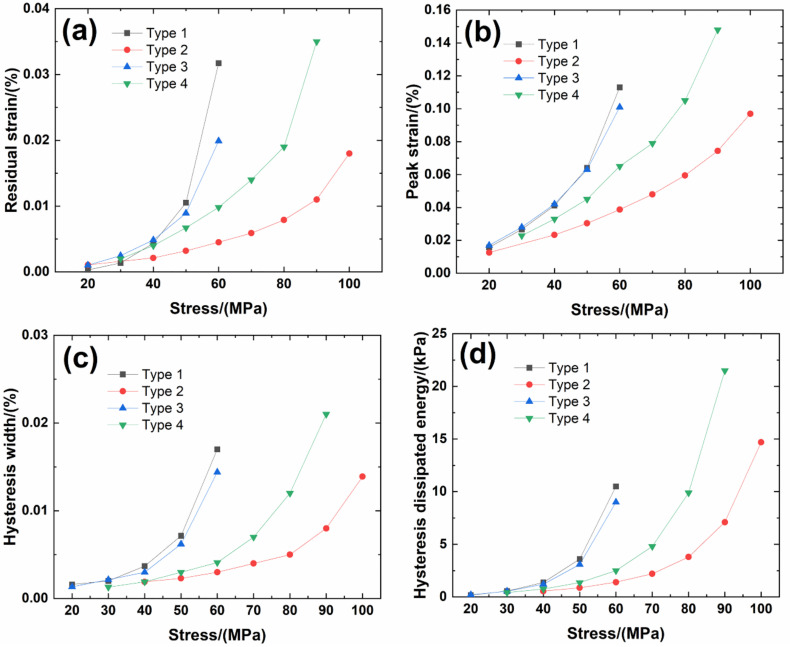
(**a**) Unloading ITM; (**b**) reloading ITM; (**c**) hysteresis width; and (**d**) hysteresis dissipated energy of Type 1 3D needle-punched C/SiC composite.

**Figure 9:**
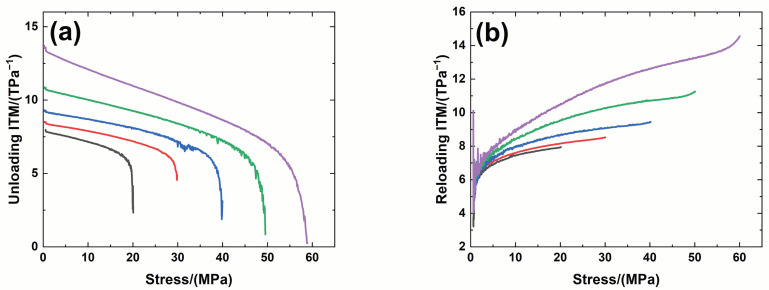
(**a**) Unloading ITM; and (**b**) reloading ITM of Type 2 3D needle-punched C/SiC composite.

**Figure 10:**
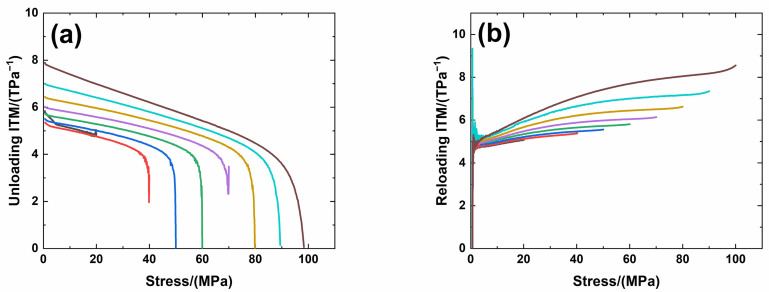
(**a**) Unloading ITM; and (**b**) reloading ITM of Type 3 3D needle-punched C/SiC composite.

**Figure 11:**
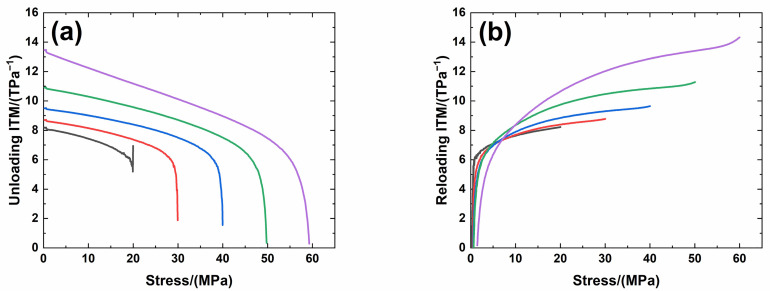
(**a**) Unloading ITM; and (**b**) reloading ITM of Type 4 3D needle-punched C/SiC composite.

**Figure 12:**
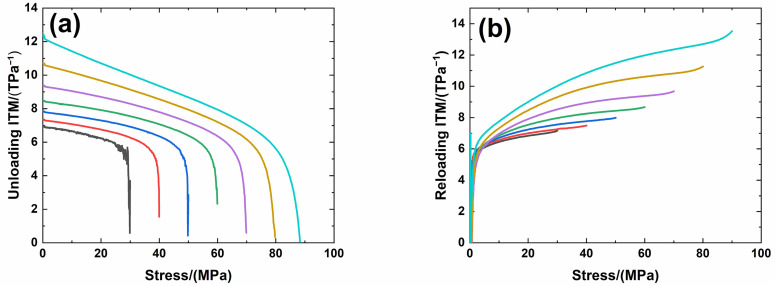
(**a**) Unloading ITM; and (**b**) reloading ITM of Type 4 3D needle-punched C/SiC composite.

Figure 6 shows experimental monotonic and cyclic loading/unloading tensile stress-strain curves of 3D needle-punched C/SiC composites with four different types of fabric preform. The tensile curves exhibit obvious non-linear appearance. Guo et al. [12] investigated the tensile fracture of 3D needled-punch C/SiC composite at room temperature, and the composite tensile strength was approximately *σ*_uts_ = 159 MPa. Under tensile loading, damage mechanisms of matrix cracking, deflection, fibers broken and pullout were observed and contributed to the nonlinear behavior of 3D needled-punch C/SiC composite. Chen et al. [14] investigated tensile behavior of 3D needled-punch C/SiC composites at room and elevated temperatures. The tensile strength increased gradually from 98.7 MPa at room temperature to 162.6 MPa at 1800 °C and then decreased to 154.3 MPa at 2000 °C. At elevated temperature, a large amount fibers were found to be pulled out at the fracture surface, indicating the decrease of the interface shear stress with temperature. Lin [27] investigated the tensile performance of needled-punch C/C composite at elevated temperature. The tensile, compressive and shear strength increased first and then decreased with the increase of temperature. Li [28] investigated monotonic and cyclic loading/unloading tensile behavior of C/SiC composite, and developed micromechanical constitutive models to predict the tensile curves and hysteresis loops. Upon unloading and reloading, the hysteresis loops appeared, which indicates the occurrence of interface debonding and slip inside of CMCs [17,29,30,31]. Figure 7 shows the composite tangent modulus versus applied stress and strain curves. The tangent modulus decreases rapidly at the initial loading stage, and then decreases gradually until final tensile fracture. Figure 8 shows the composite residual strain (ε_res_), peak strain (ε_p_), hysteresis width (Δε) and hysteresis dissipated energy (Σ) versus unloading stress curves. The unloading residual strain, reloading peak strain, hysteresis width and hysteresis dissipated energy all increases with peak stress. Vagaggnin et al. [32], Domergue et al. [33], and Guo et al. [22] investigated the ITM upon unloading and reloading for unidirectional and 2D SiC/SiC composites. Unloading and reloading ITM reflected internal damage evolution of CMCs. Upon unloading, the ITM increased rapidly first and then slowly with decreasing stress; and upon reloading, the ITM increased rapidly first and then slowly with increasing stress. The changes of ITM upon unloading and reloading were attributed to interface debonding and sliding between the fiber and the matrix. Figure 9, Figure 10, Figure 11 and Figure 12 show the ITM versus unloading and reloading stress. At the initial stage of unloading or reloading, the unloading or reloading ITM increases rapidly with decreasing or increasing stress, and then increases slowly with unloading or reloading stress.

For Type 1 3D needle-punched C/SiC composite, the tensile properties are listed in Table 2. The composite initial tangent modulus is approximately *E*_0_ = 215.6 GPa, as shown in Figure 7. When the applied stress increases to approximately *σ*_cr_ = 22 MPa, the tensile stress-strain curve begins to deflect, as shown in Figure 6a, due to damage mechanisms of matrix cracking and interface debonding, and the composite tangent modulus decreases to approximately *E* = 99.7 GPa, as shown in Figure 7. When the applied stress continued to increase, a gradual fiber fracture occurs, and the composite tensile fracture occurs at approximately *σ*_uts_ = 64.4 MPa with the failure strain of approximately *ε_f_* = 0.16%, as shown in Figure 6a. For unloading and reloading at the peak stress of *σ*_max_ = 20, 30, 40, 50 and 60 MPa, the cyclic loading/unloading damage parameters are listed in Table 3. The composite exhibits obvious hysteresis loops, as shown in Figure 6a. The composite residual strain, ε_res_, increases from ε_res_ = 0.0003% at *σ*_max_ = 20 MPa to ε_res_ = 0.03% at *σ*_max_ = 60 MPa, as shown in Figure 8a; the composite peak strain, ε_p_, increases from ε_p_ = 0.0158% at *σ*_max_ = 20 MPa to ε_p_ = 0.113% at *σ*_max_ = 60 MPa, as shown in Figure 8b; the composite hysteresis width, Δε, increases from Δε = 0.0016% at *σ*_max_ = 20 MPa to Δε = 0.017% at *σ*_max_ = 60 MPa, as shown in Figure 8c; and the composite hysteresis dissipated energy, Σ, increases from Σ = 0.18 kPa at *σ*_max_ = 20 MPa to Σ = 10.5 kPa at *σ*_max_ = 60 MPa, as shown in Figure 8d. Upon unloading at peak stress *σ*_max_ = 20 MPa, the composite unloading ITM increases rapidly from ITM = 2.3 TPa^−1^ at *σ*_max_ = 20 MPa to ITM = 6.05 TPa^−1^ at *σ* = 18.4 MPa, and then increases slowly to ITM = 7.9 TPa^−1^ at *σ* = 0.5 MPa, as shown in Figure 9a; upon reloading, the composite reloading ITM increases rapidly from ITM = 3.2 TPa^−1^ at *σ* = 0.5 MPa to ITM = 6.1 TPa^−1^ at *σ* = 1.8 MPa, and then increases slowly to ITM = 7.9 TPa^−1^ at *σ*_max_ = 20 MPa, as shown in Figure 9b. Upon unloading at peak stress *σ*_max_ = 60 MPa, the composite unloading ITM increases rapidly from ITM = 0.27 TPa^−1^ at *σ*_max_ = 60 MPa to ITM = 6.69 TPa^−1^ at *σ* = 51.7 MPa, and then increases slowly to ITM = 13.6 TPa^−1^ at *σ* = zero MPa, as shown in Figure 9a; upon reloading, the composite reloading ITM increases rapidly from ITM = 4 TPa^−1^ at *σ* = 0.6 MPa to ITM = 7.5 TPa^−1^ at *σ* = 3.7 MPa, and then increases slowly to ITM = 14.5 TPa^−1^ at *σ*_max_ = 60 MPa, as shown in Figure 9b.

For Type 2 3D needle-punched C/SiC composite, the tensile properties are listed in Table 2. The composite initial tangent modulus is approximately *E*_0_ = 220.4 GPa, as shown in Figure 7. When the applied stress increases to approximately *σ*_cr_ = 45 MPa, the tensile stress-strain curve begins to deflect, as shown in Figure 6b, due to damage mechanisms of matrix cracking and interface debonding, and the composite tangent modulus decreases to approximately *E* = 158.6 GPa, as shown in Figure 7. With increasing applied stress, more matrix cracking and interface debonding occur and the composite tangent modulus decreases slowly with applied stress, as shown in Figure 7. The composite fractures at approximately *σ*_uts_ = 112.3 MPa with the fracture strain of approximately *ε_f_* = 0.25%, as shown in Figure 6b. Upon unloading and reloading at the peak stress of *σ*_max_ = 20, 40, 50, 60, 70, 80, 90 and 100 MPa, the cyclic loading/unloading related damage parameters are listed in Table 4. The composite exhibits obvious hysteresis loops, as shown in Figure 6b. The composite residual strain, *ε*_res_, increases from ε_res_ = 0.001% at *σ*_max_ = 20 MPa to *ε*_res_ = 0.018% at *σ*_max_ = 100 MPa, as shown in Figure 8a; the composite peak strain, *ε*_p_, increases from ε_p_ = 0.0126% at *σ*_max_ = 20 MPa to ε_p_ = 0.097% at *σ*_max_ = 100 MPa, as shown in Figure 8b; the composite hysteresis width, Δε, increases from Δε = 0.0019% at *σ*_max_ = 40 MPa to Δε = 0.0139% at *σ*_max_ = 100 MPa, as shown in Figure 8c; and the composite hysteresis dissipated energy, Σ, increases from Σ = 0.549 kPa at *σ*_max_ = 40 MPa to Σ = 14.7 kPa at *σ*_max_ = 100 MPa, as shown in Figure 8d. Upon unloading at peak stress *σ*_max_ = 40 MPa, the composite unloading ITM increases rapidly from ITM = 1.95 TPa^−1^ at *σ*_max_ = 40 MPa to ITM = 4.09 TPa^−1^ at *σ* = 36 MPa, and then increases slowly to ITM = 5.3 TPa^−1^ at *σ* = 0.6 MPa, as shown in Figure 10a; upon reloading, the composite reloading ITM increases rapidly from ITM = 2.3 TPa^−1^ at *σ* = 0.6 MPa to ITM = 4.75 TPa^−1^ at *σ* = 2.9 MPa, and then increases slowly to ITM = 5.38 TPa^−1^ at *σ*_max_ = 40 MPa, as shown in Figure 10b. Upon unloading at peak stress *σ*_max_ = 100 MPa, the composite unloading ITM increases rapidly from ITM = 0.05 TPa^−1^ at *σ*_max_ = 100 MPa to ITM = 4.1 TPa^−1^ at *σ* = 84 MPa, and then increases slowly to ITM = 7.9 TPa^−1^ at *σ* = 0.5 MPa, as shown in Figure 10a; upon reloading, the composite reloading ITM increases rapidly from ITM = 1.4 TPa^−1^ at *σ* = 0.66 MPa to ITM = 4.9 TPa^−1^ at *σ* = 2.5 MPa, and then increases slowly to ITM = 8.5 TPa^−1^ at *σ*_max_ = 100 MPa, as shown in Figure 10b.

For Type 3 3D needle-punched C/SiC composite, the tensile properties are listed in Table 2. The composite initial tangent modulus is approximately *E*_0_ = 161.4 GPa, as shown in Figure 7. When the applied stress increases to approximately *σ*_cr_ = 20 MPa, the tensile stress-strain curve begins to deflect, as shown in Figure 6c, due to damage mechanisms of matrix cracking and interface debonding, and the composite tangent modulus decreases to approximately *E* = 97.7 GPa, as shown in Figure 7. When the applied stress continues to increase, more damages of matrix cracking and interface debonding occur, and some fibers begin to fracture. The composite fracture occurs at approximately *σ*_uts_ = 67.5 MPa with the failure strain of approximately *ε*_f_ = 0.168%, as shown in Figure 6c. Upon unloading and reloading at the peak stress of *σ*_max_ = 20, 30, 40, 50 and 60 MPa, the cyclic loading/unloading damage parameters are listed in Table 5. The composite exhibits obvious hysteresis loops, as shown in Figure 6c. The composite residual strain, ε_res_, increases from *ε*_res_ = 0.001% at *σ*_max_ = 20 MPa to *ε*_res_ = 0.019% at *σ*_max_ = 60 MPa, as shown in Figure 8a; the composite peak strain, *ε*_p_, increases from *ε*_p_ = 0.017% at *σ*_max_ = 20 MPa to *ε*_p_ = 0.101% at *σ*_max_ = 60 MPa, as shown in Figure 8b; the composite hysteresis width, Δε, increases from Δε = 0.0013% at *σ*_max_ = 20 MPa to Δε = 0.0144% at *σ*_max_ = 60 MPa, as shown in Figure 8c; and the composite hysteresis dissipated energy, Σ, increases from Σ = 0.196 kPa at *σ*_max_ = 20 MPa to Σ = 9 kPa at *σ*_max_ = 60 MPa, as shown in Figure 8d. Upon unloading at peak stress *σ*_max_ = 20 MPa, the composite unloading ITM increases rapidly from ITM = 5.17 TPa^−1^ at *σ*_max_ = 20 MPa to ITM = 6.19 TPa^−1^ at *σ* = 18.6 MPa, and then increases slowly to ITM = 8.2 TPa^−1^ at *σ* = 0.2 MPa, as shown in Figure 11a; upon reloading, the composite reloading ITM increases rapidly from ITM = 1.1 TPa^−1^ at *σ* = 0.3 MPa to ITM = 6.1 TPa^−1^ at *σ* = 0.9 MPa, and then increases slowly to ITM = 8.22 TPa^−1^ at *σ*_max_ = 20 MPa, as shown in Figure 11b. Upon unloading at peak stress *σ*_max_ = 60 MPa, the composite unloading ITM increases rapidly from ITM = 0.28 TPa^−1^ at *σ*_max_ = 60 MPa to ITM = 5.97 TPa^−1^ at *σ* = 55.1 MPa, and then increases slowly to ITM = 13.4 TPa^−1^ at *σ* = 0.4 MPa, as shown in Figure 11a; upon reloading, the composite reloading ITM increases rapidly from ITM = 0.16 TPa^−1^ at *σ* = 1.4 MPa to ITM = 6.93 TPa^−1^ at *σ* = 6 MPa, and then increases slowly to ITM = 14.3 TPa^−1^ at *σ*_max_ = 60 MPa, as shown in Figure 11b.

For Type 4 3D needle-punched C/SiC composite, the tensile properties are listed in Table 2. The composite initial tangent modulus is approximately *E*_0_ = 178.2 GPa, as shown in Figure 7. When the applied stress increases to approximately *σ*_cr_ = 35 MPa, the tensile stress–strain curve begins to deflect, as shown in Figure 6d, due to damage mechanisms of matrix cracking and interface debonding, and the composite tangent modulus decreases to approximately *E* = 98.2 GPa, as shown in Figure 7. When the applied stress continues to increase, more damage in terms of matrix cracking and interface debonding occur, and some fibers begin to fracture. The composite fracture occurs at approximately *σ*_uts_ = 101.3 MPa with the failure strain of approximately *ε*_f_ = 0.2%, as shown in Figure 6d. Upon unloading and reloading at the peak stress of *σ*_max_ = 30, 40, 50, 60, 70, 80 and 90 MPa, the cyclic loading/unloading damage parameters are listed in Table 6. The composite exhibits obvious hysteresis loops, as shown in Figure 6d. The composite residual strain, *ε*_res_, increases from *ε*_res_ = 0.002% at *σ*_max_ = 30 MPa to ε_res_ = 0.035% at *σ*_max_ = 90 MPa, as shown in Figure 8a; the composite peak strain, ε_p_, increases from ε_p_ = 0.0228% at *σ*_max_ = 30 MPa to *ε*_p_ = 0.148% at *σ*_max_ = 90 MPa, as shown in Figure 8b; the composite hysteresis width, Δε, increases from Δε = 0.0013% at *σ*_max_ = 30 MPa to Δε = 0.021% at *σ*_max_ = 90 MPa, as shown in Figure 8c; and the composite hysteresis dissipated energy, Σ, increases from Σ = 0.418 kPa at *σ*_max_ = 30 MPa to Σ = 21.5 kPa at *σ*_max_ = 90 MPa, as shown in Figure 8d. Upon unloading at peak stress *σ*_max_ = 30 MPa, the composite unloading ITM increases rapidly from ITM = 0.58 TPa^−1^ at *σ*_max_ = 30 MPa to ITM = 5.21 TPa^−1^ at *σ* = 28 MPa, and then increases slowly to ITM = 6.96 TPa^−1^ at *σ* = 0.26 MPa, as shown in Figure 12a; upon reloading, the composite reloading ITM increases rapidly from ITM = 2.38 TPa^−1^ at *σ* = 0.4 MPa to ITM = 5.9 TPa^−1^ at *σ* = 3.9 MPa, and then increases slowly to ITM = 7.2 TPa^−1^ at *σ*_max_ = 30 MPa, as shown in Figure 12b. Upon unloading at peak stress *σ*_max_ = 90 MPa, the composite unloading ITM increases rapidly from ITM = 0.05 TPa^−1^ at *σ*_max_ = 90 MPa to ITM = 5.42 TPa^−1^ at *σ* = 80 MPa, and then increases slowly to ITM = 12.5 TPa^−1^ at *σ* = zero MPa, as shown in Figure 12a; upon reloading, the composite reloading ITM increases rapidly from ITM = 1.43 TPa^−1^ at *σ* = 0.07 MPa to ITM = 6.5 TPa^−1^ at *σ* = 2.9 MPa, and then increases slowly to ITM = 13.5 TPa^−1^ at *σ*_max_ = 90 MPa, as shown in Figure 12b.

For Type 1–4 3D needle-punched C/SiC composites, the composite initial tangent modulus, tensile strength and facture strain are the highest, the residual strain, peak strain, hysteresis width and hysteresis dissipated energy are the lowest for Type 2 needle-punched C/SiC composite, due to the highest fiber volume fraction along the loading direction, as shown in Table 1. The composite tensile strength and fracture strain are the lowest, and the residual strain, peak strain, hysteresis width and hysteresis dissipated energy are the highest for Type 1 needle-punched C/SiC composite, due to the lowest fiber volume fraction along the loading direction, as shown in Table 1. When the fiber volume fraction along the loading direction increases, the stress carried by the longitudinal fibers increases, and the interface debonding length decreases, which decreases the residual strain, peak strain, hysteresis width and hysteresis loops area.

**Figure 13:**
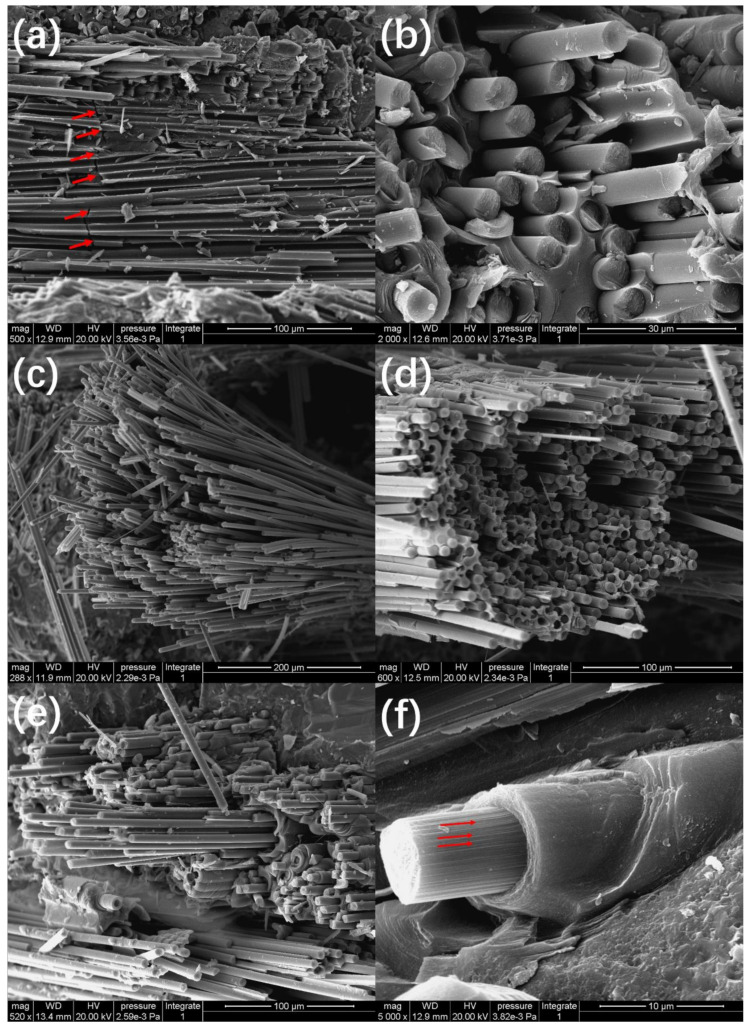
(**a**) Matrix cracking; (**b**) fibers pull out from the twill woven ply; (**c**) fibers pull out from the [45°] ply; (**d**) fibers pull out from the [−45°] ply; (**e**) fibers pull out from the [±45°] plies; and (**f**) the single pulled out fiber.

Under monotonic and cyclic loading/unloading tensile, the non-linear tensile strain and loading/unloading hysteresis loops are mainly attributed to micro damage mechanisms of matrix cracking (Figure 13a), interface debonding (Figure 13b,f), and fibers pullout (Figure 13b–f). When matrix cracking, interface debonding, fibers fracture and pullout occur, fiber sliding relative to the matrix in the interface debonding region, leading to the increase of loading/unloading residual strain, peak strain, hysteresis loops width and hysteresis loops area. For twill woven ply, the fiber pullout length is short (Figure 13b) and the fiber pullout length is long for [±45°] plies (Figure 13c–e). There are obvious scratches on the surface of the pullout fibers due to interface frictional sliding, as shown in Figure 13f.

## 4. Summary and Conclusions

In this paper, monotonic and cyclic loading/unloading tensile behavior of four different 3D needle-punched C/SiC composites were investigated. Under tensile loading, tensile tangent modulus, tensile strength and fracture strain were used to characterize tensile behavior. Under cyclic loading/unloading, multiple damage parameters of unloading residual strain, tensile peak strain, hysteresis loops width, hysteresis loops area, unloading and reloading ITM were adopted to characterize the damage evolution subjected to tensile loading. After tensile fracture, the fracture surfaces were observed under a SEM. Damage in terms of matrix cracking, interface debonding, and fiber fracture and pullout in different plies was observed. Relationships between composite tensile mechanical behavior, micro damage parameters, and micro damage mechanisms were established.

(1) For Types 1–4 3D needle-punched C/SiC composite, the composite initial tangent modulus was in the range of 161.4–220.4 GPa, the composite tensile strength was in the range of 64.4–112.3 MPa, and the composite fracture strain was in the range of 0.16–0.25%.
(2) Under monotonic and cyclic loading/unloading tensile, the 3D needled-punch C/SiC composites exhibited obviously non-linear and hysteresis loops behavior. The composite tensile and hysteresis behavior depended on the fiber volume fraction along the loading direction. For Type 2 C/SiC composite with the high fiber volume fraction along the loading direction, the composite tensile strength and fracture strain were the highest, and the residual strain, peak strain, hysteresis width, and hysteresis area were the lowest among the four types of C/SiC composites. Upon increasing peak stress from *σ*_max_ = 40 to 100 MPa, the composite residual strain increases from ε_res_ = 0.001% to 0.018%, the peak strain increases from ε_p_ = 0.0126% to 0.097%, the hysteresis loops width increases from Δε = 0.0019% to 0.0139%, and the hysteresis dissipated energy increases from Σ = 0.549 kPa to 14.7 kPa.
(3) At the fracture surface, the fiber pullout lengths on the twill woven plies were short, and the lengths of fiber pullout on the [±45°] plies were long.

## Figures and Tables

**Table 1:** Composite fabric raw materials and woven structural parameters.

Num	Fabric Preform	Fabric Forming Method	Density of Original Fabric /(kg/m^3^)	Fiber Volume of Original Fabric/(%)	Density of Heat-Treated Fabric/(kg/m^3^)	Fiber Volume of Heat-Treated Fabric/(%)
1#	HTSTM-3K twill woven cloth/ T700^TM^-12K [±45°] plain woven cloth/T700^TM^-12K short-chopped-fiber web	Needle	540	30	460	25.6
2#	HTSTM-3K twill woven ply/T700^TM^-12K [0°] non-woven cloth/T700^TM^-12K [±45°] plain woven cloth/T700^TM^-12K short-chopped-fiber web	Needle	670	37.2	590	32.8
3#	Two plies of HTS^TM^-3K twill woven cloth/T700^TM^ [±45°] plain woven cloth/T700TM-12K short-chopped-fiber web	Needle	680	37.8	470	26.1
4#	Two plies of HTS^TM^-3K twill woven cloth/T700^TM^-12K [±45°] plain woven cloth/T700^TM^-12K short-chopped-fiber web	Needle + Stitch	670	37.2	580	32.2

**Table 2:** Tensile properties of Types 1–4 3D needle-punched C/SiC composites.

Type 1		Type 2		Type 3		Type 4	
*E*_0_/(GPa)	215.6	*E*_0_/(GPa)	220.4	*E*_0_/(GPa)	161.4	*E*_0_/(GPa)	178.2
*σ*_cr_/(MPa)	22	*σ*_cr_/(MPa)	45	*σ*_cr_/(MPa)	20	*σ*_cr_/(MPa)	35
*σ*_uts_/(MPa)	64.4	*σ*_uts_/(MPa)	112.3	*σ*_uts_/(MPa)	67.5	*σ*_uts_/(MPa)	101.3
*ε*_f_/(%)	0.16	*ε*_f_/(%)	0.25	*ε*_f_/(%)	0.168	*ε*_f_/(%)	0.2

**Table 3:** Cyclic loading/unloading damage parameters of Type 1 3D needle-punched C/SiC composite.

Unloading Stress/(MPa)	ε_res_/(%)	ε_p_/(%)	Δε/(%)	Σ/(kPa)
20	0.0003	0.0158	0.0016	0.18
30	0.00136	0.0266	0.002	0.55
40	0.00435	0.0411	0.00369	1.38
50	0.01052	0.064	0.00715	3.6
60	0.03173	0.113	0.017	10.5

**Table 4:** Cyclic loading/unloading damage parameters of Type 2 3D needle-punched C/SiC composite.

Unloading Stress/(MPa)	ε_res_/(%)	ε_p_/(%)	Δε/(%)	Σ/(kPa)
20	0.00106	0.0126	-	-
40	0.00213	0.0233	0.0019	0.549
50	0.0032	0.0304	0.0023	0.867
60	0.0045	0.0387	0.003	1.4
70	0.0059	0.048	0.004	2.2
80	0.0079	0.0595	0.005	3.8
90	0.011	0.0744	0.008	7.1
100	0.018	0.097	0.0139	14.7

**Table 5:** Cyclic loading/unloading damage parameters of Type 3 3D needle-punched C/SiC composite.

Unloading Stress/(MPa)	ε_res_/(%)	ε_p_/(%)	Δε/(%)	Σ/(kPa)
20	0.00103	0.017	0.00133	0.196
30	0.00247	0.028	0.00215	0.539
40	0.00484	0.042	0.003	1.21
50	0.00893	0.063	0.0062	3.09
60	0.0199	0.101	0.0144	9

**Table 6:** Cyclic loading/unloading damage parameters of Type 4 3D needle-punched C/SiC composite.

Unloading Stress/(MPa)	ε_res_/(%)	ε_p_/(%)	Δε/(%)	Σ/(kPa)
30	0.002	0.0228	0.0013	0.418
40	0.004	0.033	0.0019	0.752
50	0.0067	0.045	0.003	1.36
60	0.0098	0.065	0.0041	2.5
70	0.014	0.079	0.007	4.8
80	0.019	0.105	0.012	9.9
90	0.035	0.148	0.021	21.5

## Data Availability

The data used to support the findings of this study are available from the paper.
